# Omega‐3 supplementation attenuates doxorubicin‐induced cardiotoxicity but is not related to the ceramide pathway

**DOI:** 10.1002/fsn3.4492

**Published:** 2024-09-29

**Authors:** Marina Gaiato Monte, Carolina Rodrigues Tonon, Anderson Seiji Fujimori, Ana Paula Dantas Ribeiro, Silmeia Garcia Zanati, Katashi Okoshi, Camila Renata Correa Camacho, Maria Regina Moretto, Sergio Alberto Rupp de Paiva, Leonardo Antonio Mamede Zornoff, Paula Schmidt Azevedo, Marcos Ferreira Minicucci, Bertha Furlan Polegato

**Affiliations:** ^1^ Department of Internal Medicine Botucatu Medical School, São Paulo State University‐UNESP Botucatu Brazil; ^2^ Department of Pathology Botucatu Medical School, São Paulo State University‐UNESP Botucatu Brazil; ^3^ Experimental Research Unit Botucatu Medical School, São Paulo State University‐UNESP Botucatu Brazil

**Keywords:** chronic cardiotoxicity, doxorubicin, dyslipidemia, omega‐3 fatty acids, oxidative stress, sphingomyelins

## Abstract

Cardiotoxicity is the serious side effect of doxorubicin treatment. Ceramides are formed from the degradation of sphingolipids in cell membranes and play an important role in signaling and modulating biological processes. There is evidence that omega‐3 fatty acid administration can act on this pathway. To evaluate the role of the ceramide pathway in the pathophysiology of doxorubicin‐induced cardiotoxicity and the effect of omega‐3 fatty acid supplementation in the attenuation of chronic doxorubicin‐induced cardiotoxicity in rats. Sixty male Wistar rats were divided into four groups: Control (C), Doxorubicin (D), Omega‐3 fatty acids (W), and Doxorubicin + Omega‐3 fatty acids (DW). The groups received omega‐3 fatty acids (400 mg/kg/day, via gavage) or water for 6 weeks and doxorubicin (3.5 mg/kg, intraperitoneal) or saline once a week for 4 weeks. Doxorubicin‐treated animals showed increases in left atrium and left ventricle diameters, serum triglycerides and cholesterol, malondialdehyde, and protein carbonylation. We also observed a decrease in left ventricular shortening fraction and nSMase1 expression in the heart. Omega‐3 fatty acid supplementation attenuated the structural and functional alterations caused by doxorubicin and decreased protein carbonylation. In contrast to doxorubicin, omega‐3 fatty acids increased neutral nSMase activity in animals that both received and did not receive doxorubicin but with no effect on nSMase1 protein expression. Omega‐3 fatty acid supplementation attenuated the cardiotoxicity caused by doxorubicin. The ceramide pathway may be involved in the pathophysiology of cardiotoxicity, but it is not the mechanism by which omega‐3 fatty acids attenuated cardiac dysfunction.

## Graphical abstract

This is experimental research that showed the effect of omega‐3 fatty acids administration on cardiac toxicity induced by doxorubicin. Interestingly, the omega‐3 mitigated the deleterious effect of doxorubicin in the heart, but it did not affect the ceramide pathway, that was our original hypothesis. DOXO: doxorubicin; EPA: eicosapentaenoic acid; DHA: docosahexaenoic acid; LVFS: LV fraction shortening; LAD: left atrium diameter; LV: left ventricle; MDA: malondialdehyde; nSMases: neutral sphingomyelinase; TG: triglycerides.

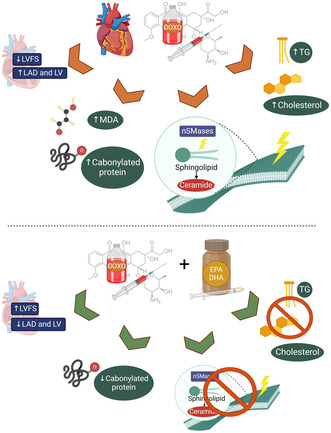

## 1 INTRODUCTION

Cancer is one of the leading causes of mortality worldwide. In 2023, it was estimated that 609,820 people would die from cancer in the USA, corresponding to approximately 1670 deaths per day (Siegel et al., 2023). Advances in cancer therapy have improved patient quality of life and survival, but heart disease is frequently reported in these patients due to the side effects of chemotherapy drugs (Ghigo et al., 2016).

Doxorubicin is a highly effective chemotherapy drug used to treat breast, lung, and hematological cancers; however, it comes with the risk of cardiotoxicity. Chronic cardiotoxicity, which occurs weeks or months after drug infusion, is dose dependent. A cumulative dose of 250–400 mg/m^2^ of doxorubicin can cause left ventricular dysfunction between 25% and 35% of patients (Rawat et al., 2021).

Several studies have highlighted the role of oxidative stress in the development of doxorubicin‐induced cardiotoxicity. Excessive reactive oxygen (ROS) and reactive nitrogen (RNS) species production during drug metabolism, associated with low myocardial antioxidant enzyme concentrations may interfere with cardiac tissue damage. Also, doxorubicin can cause mitochondrial dysfunction by interacting with mitochondrial membranes, which have a great quantity of cardiolipin and high affinity to the drug, causing interruption of the electron transport chain and accumulation of unpaired electrons, thus increasing ROS formation (Lopaschuk et al., 2021; Russo et al., 2021).

Despite the central role of oxidative stress in this context, antioxidant therapies have shown little effect as cardio‐protectors against doxorubicin toxicity. Therefore, other pathophysiological pathways have been investigated as potential targets for therapeutic interventions (van Dalen et al., 2008).

The ceramide pathway is already known in the pathophysiology of cardiovascular disease but has not been well explored, especially in doxorubicin‐induced cardiotoxicity (Choi et al., 2021; Kretzschmar et al., 2021; Yun et al., 2021). Cell membranes are formed by cholesterol, sphingolipids, and other phospholipids. The major component of a sphingolipid is sphingomyelin which is degraded by sphingomyelinases (SMases) in situations where there is physical or chemical aggression, such as the use of chemotherapy drugs. Sphingomyelin breakdown generates ceramide, which acts in the regulation of inflammation, cell proliferation and differentiation, oxidative homeostasis, and apoptosis (Choi et al., 2021; Taniguchi & Okazaki, 2014). The increase in cellular oxidative stress induced by ceramide is related to NADPH oxidase activation, mitochondrial dysfunction, and a decrease in antioxidant enzymes (Won & Singh, 2006). Another pathway possibly involved in the deleterious role of ceramides is the activation of NF‐kB (nuclear factor kappa B) mediated by TNF (tumor necrosis factor) (Al‐Rashed et al., 2020; Yang & Jiang, 2019). Additionally, ceramide‐induced lipotoxicity plays a crucial role in the development of cardiovascular diseases. Ceramides drive metabolic changes that manage excess lipids by enhancing fatty acid uptake and reducing glucose utilization through inhibition of Akt/PKB activation. Over time, ceramides trigger apoptosis and fibrosis to eliminate damaged cells, but chronic activation of these processes can contribute to the onset of cardiovascular disease (Summers, 2021; Wang et al., 2017; Zietzer et al., 2022).

Eicosapentaenoic acid (EPA) and docosahexaenoic acid (DHA) present in cell membranes are long‐chain polyunsaturated fatty acids of the omega‐3 family. These can be used as substrates for the formation of anti‐inflammatory eicosanoids by the lipoxygenase and cyclooxygenase pathways (Calder, 2012), which suggests that omega‐3 fatty acid could have a beneficial effect in the context of cardiotoxicity.

In fact, an in vitro study which added omega‐3 fatty acids to doxorubicin‐treated cardiomyocytes decreased expression of inflammatory cytokines (TNFα, interleukin 1β and interleukin 6) and inhibited activation of the NF‐kB pathway (Wang et al., 2016). In vivo studies with cardiotoxicity models associated with omega‐3 fatty acid treatment have shown a reduction in malondialdehyde, a marker of lipid peroxidation, and an increase in antioxidant enzyme activity (El Amrousy et al., 2022; de Barros et al., 2021; Saleh et al., 2020; Uygur et al., 2014).

Additionally, there is evidence that exogenous supplementation of omega‐3 fatty acids may act in the ceramide formation pathway. In cell culture, DHA supplementation decreased oxidative stress and ceramide formation after LPS‐stimulated inflammation (Jin et al., 2018). Supplementation of EPA and DHA together reduced the synthesis of ceramides in the liver and muscles of hamsters fed a high‐fat diet, culminating in an increase in glucose tolerance and a decrease in hepatic triglyceride secretion (Kasbi‐Chadli et al., 2016).

Also, EPA and DHA supplementation showed possible beneficial effects on cardiovascular events such as a decrease in atherosclerotic plaque and normalization of plasma triglycerides. However, these effects are still contradictory (Backes et al., 2016; Colussi et al., 2017).

In the context of in vivo studies on doxorubicin‐induced cardiotoxicity and omega‐3 fatty acids, research by Saleh et al. (2020) and Uygur et al. (2014) demonstrated that omega‐3 fatty acid supplementation reduced malondialdehyde (MDA) levels and enhanced the activity of antioxidant enzymes, including glutathione peroxidase (GSH‐Px) and superoxide dismutase (SOD), in rats with acute doxorubicin‐induced cardiotoxicity. Similarly, El Amrousy et al. (2022) observed comparable improvements in serum markers in children with leukemia receiving doxorubicin when supplemented with omega‐3 fatty acids. Given these positive findings, along with the involvement of omega‐3 fatty acids in the ceramide pathway and the complexities of the pathophysiology of doxorubicin‐induced cardiotoxicity, this study aimed to investigate the role of the ceramide pathway in this condition and evaluate the potential of omega‐3 fatty acid supplementation to mitigate chronic doxorubicin‐induced cardiotoxicity in rats.

## 2 MATERIALS AND METHODS

### 2.1 Sample size calculation

Left ventricle shortening fraction obtained from previous studies was used to calculate sample size. Considering four experimental groups, a mean difference of 0.09, a standard deviation of 0.06, a test power of 0.90, and an alpha error of 0.05, we obtained a sample size of 14 animals per group. Also, mortality in our previous studies during chronic doxorubicin treatment ranged between 10 and 20%, so we added two animals to each doxorubicin‐treated group.

### 2.2 Study design

This study received prior approval from our local ethics committee (protocol 1295/2019) following the Brazilian National Council standards for the Control of Animal Experimentation. Sixty male Wistar rats, weighing 280 ± 25 g, were kept in a controlled enriched environment with a 12‐h light/dark cycle at 23 ± 2°C and free access to chow and filtered water. Animal weight and chow intake were measured weekly. Rats were divided into four groups: Control (C; *n* = 14), Omega‐3 fatty acids (W; n = 14), Doxorubicin (D; *n* = 16), and Doxorubicin + Omega‐3 fatty acids (DW; n = 16). Groups W and DW received 400 mg/kg omega‐3 fatty acids daily by intragastric gavage (IG) for 6 weeks. Groups C and D received an equivalent volume of filtered water (IG). The omega‐3 fatty acid supplement used in this study was Super Omega‐3 TG® by Essential Nutrition; each capsule (1000 mg) contained EPA (360 mg), DHA (240 mg), other fatty acids (350 mg), and DL‐alpha‐tocopherol (1.6 mg). Two weeks after beginning omega‐3 fatty acid supplementation, doxorubicin administration was initiated in the D and DW groups by intraperitoneal (IP) injection (3.5 mg/kg), once a week for 4 weeks (total dosage: 14 mg/kg). C and W groups received an equivalent volume of saline IP at the same frequency. After 6 weeks, all animals were sedated for echocardiographic analysis and were then sacrificed with sodium thiopental (120 mg/kg, IP). Blood was collected and centrifuged, and serum was stored at −80°C. Hearts were dissected, weighed, and stored at −80°C.

### 2.3 Echocardiogram

Animals were anesthetized with ketamine (50 mg/kg, IP) and xylazine (1 mg/kg, IP) and placed in supine position. Echocardiogram was performed with a GE Vivid S6 system (General Electric Medical Systems) by an examiner blinded to animal group. Structural variables such as posterior wall thickness, interventricular septum thickness, left atrium diameter, aorta diameter, and left ventricle diameter in systole (LVSD) and diastole (LVDD) were obtained. Left atrium/aorta diameter ratio was calculated. Isovolumetric relaxation time (IVRT), E‐wave deceleration time (EDT), peak early diastolic filling velocity (E wave), and peak late diastolic filling velocity (A wave) were obtained by transmitral Doppler. Systolic (S′), early diastolic (E′), and late diastolic (A′) displacements of the mitral ring in the lateral and septal wall were also obtained by tissue Doppler. Systolic function was analyzed by left ventricular shortening fraction [(LVDD−LVSD/LVDD)] and S′. Diastolic function was analyzed by E wave, A wave, IVRT, EDT, and E/A and E/E′ ratios.

### 2.4 Serum lipid profile and serum creatinine

We measured serum creatinine, total cholesterol (TC), high‐density lipoprotein (HDL), and triglyceride (TG) concentrations. Measurements were performed by enzymatic colorimetric method using a commercial kit (Bioclin, Minas Gerais, Brazil).

### 2.5 Neutral sphingomyelinase activity assay

Neutral sphingomyelinase activity was determined in serum and left ventricular tissue homogenate. Left ventricular extraction was performed with radioimmunoprecipitation assay (RIPA) buffer and serum in 1:2 concentration. The assay allowed enzymatic activity analysis by converting the phosphocholine generated by sphingomyelinase into choline with alkaline phosphatase, choline betaine, and H_2_O_2_ with choline oxidase. H_2_O_2_ plus horseradish peroxidase act to oxidize non‐fluorescent Amplex Red to fluorescent resorufin. Amplex Red commercial kit (Amplex™ Red Sphingomyelinase Assay Kit, cat. n° A12220, Invitrogen, Waltham, MA, USA) was used for this analysis which was performed according to manufacturer's instructions.

### 2.6 Oxidative stress

Lipid peroxidation was determined by MDA (malondialdehyde) concentration in animal heart tissue using the TBARS (Thiobarbituric acid‐reactive substances) method described by Nielsen et al. (1997). For quantification, 200 μL of supernatant from the homogenate of each sample was diluted with 500 μL of solution (0.67% TBA, 10% TCA, 0.25 N HCl) and centrifuged for 5 min at 10,000 rpm. Samples were then incubated in a water bath at approximately 100°C for 45 min and subsequently transferred to a microplate (200 μL). Readings were performed at 532 and 600 nm on a Spectra Max 190 microplate reader (Molecular Devices®, Sunnyvale, CA, USA). Results were expressed as MDA‐TBA2 concentration [μmol/L] per milligram (mg) of protein (Nielsen et al., 1997).

Carbonylation was analyzed using the method adapted from Mesquita et al. (2014) where 2,4‐dinitrophenylhydrazine (DNPH) (10 mM in 2 M HCl) was added to 100 μL of cardiac tissue supernatant. Samples were incubated for 10 min at room temperature, and then 50 μL of NaOH (6 M) was added and incubated for another 10 min at room temperature. Readings were carried out at 450 nm on a Spectra Max 190 microplate reader (Molecular Devices®, Sunnyvale, CA, USA), and the results obtained were sample absorbance and molar extinction coefficient (22,000 M^−1^ cm^−1^). The final result was expressed in nmol/mg of proteins (Mesquita et al., 2014).

### 2.7 Western blot

Samples from the left ventricle (50 mg) were homogenized in RIPA buffer and centrifuged. The resulting homogenates were collected, and total protein concentrations were determined by the Bradford method. Samples were mixed with Laemmli buffer (Sigma Aldrich, San Luis, MO, USA), heated, and applied to acrylamide gel for electrophoresis. After electrophoresis, the proteins were transferred to nitrocellulose membrane and incubated with 5% skimmed milk blocking for 1 h. Afterward, the membrane was incubated with nSMase1 primary antibody (ab‐131330, ABCAM, Cambridge, UK), total IkBα (sc‐1643, Santa Cruz Biotechnology, Dallas, TX, USA), and phosphorylated IkBα (sc‐8404, Santa Cruz Biotechnology, Dallas, TX, USA) primary antibody, diluted in TBS / 3% skimmed milk overnight. The membrane was washed three times with TBS, and the secondary antibody was incubated in TBS/1% skimmed milk. The membrane was washed three more times, and immunodetection was performed by chemiluminescence in an ImageQuant LAS camera imaging system (General Electrics, Boston, MD, USA). Images analysis was performed with Gel‐Pro 32 (Media Cybernetics Rockville, MD, USA) and GAPDH (mouse monoclonal IgG, sc‐32233, Santa Cruz Biotechnology, Dallas, TX, USA) used as housekeeping.

### 2.8 Immunohistochemistry

This analysis was used to identify nSMase1 in cardiac tissue. For this, cross‐sectional slices of the left ventricle were obtained, incubated in formalin, and subsequently embedded in a paraffin block until the moment of analysis. After deparaffinization and rehydration with xylene and ethyl alcohol, antigenic recovery was performed using citrate buffer pH 6.0 in a pressure cooker for 20 min. Slides were then washed with distilled water, and endogenous peroxidase (Hydrogen Peroxide Block, Spring Bioscience, California, USA) incubation was performed for 30 min at room temperature. Sections were washed with PBS, and protein blocking (Protein Block, Spring Bioscience, Pleasanton, CA, USA) was performed with 30 minutes of incubation at room temperature. Primary antibody (nSMase1/ab‐131330 ABCAM, Cambridge, UK) was incubated overnight at 4°C. Slides were washed with PBS for secondary antibody (anti‐mouse and anti‐rabbit primary antibodies, Histofine® Simple Stain MAX PO, Tokyo, Japan) incubation (30 min at room temperature). After washes with PBS, DAB chromogen (diaminobenzidine, Scytek Laboratories, Logan, UT, USA) was incubated for 5 min and washed again. Finally, hematoxylin counterstaining was performed, and slides were dehydrated with xylene and alcohol. Images were captured at 40× magnification by microscope and quantified by the ImageJ program.

### 2.9 Statistical analyses

Variables with normal distribution are presented as means ± SD. Variables with non‐normal distribution were normalized by mathematical transformation and then presented as means ± SD. Statistical analyses were performed by two‐way analysis of variance followed by the Holm‐Sidak test. When interaction between two factors occurred (doxorubicin and omega‐3 fatty acid intake), we compared groups of interest (CxD, CxW, WxDW, and DxDW). When interaction between the two factors did not occur, marginal data were presented (doxorubicin and omega‐3 fatty acid effect isolated). A statistical significance of *p* < .05 was adopted for all analyses. Variables that could not be normalized by mathematical transformation were evaluated by the *t* or Mann–Whitney test, and the *p*‐value was corrected by Bonferroni (significance if *p* < .008).

## 3 RESULTS

During the experiment, only one animal from D and one animal from DW died.

### 3.1 Body weight and food intake

**FIGURE 1:**
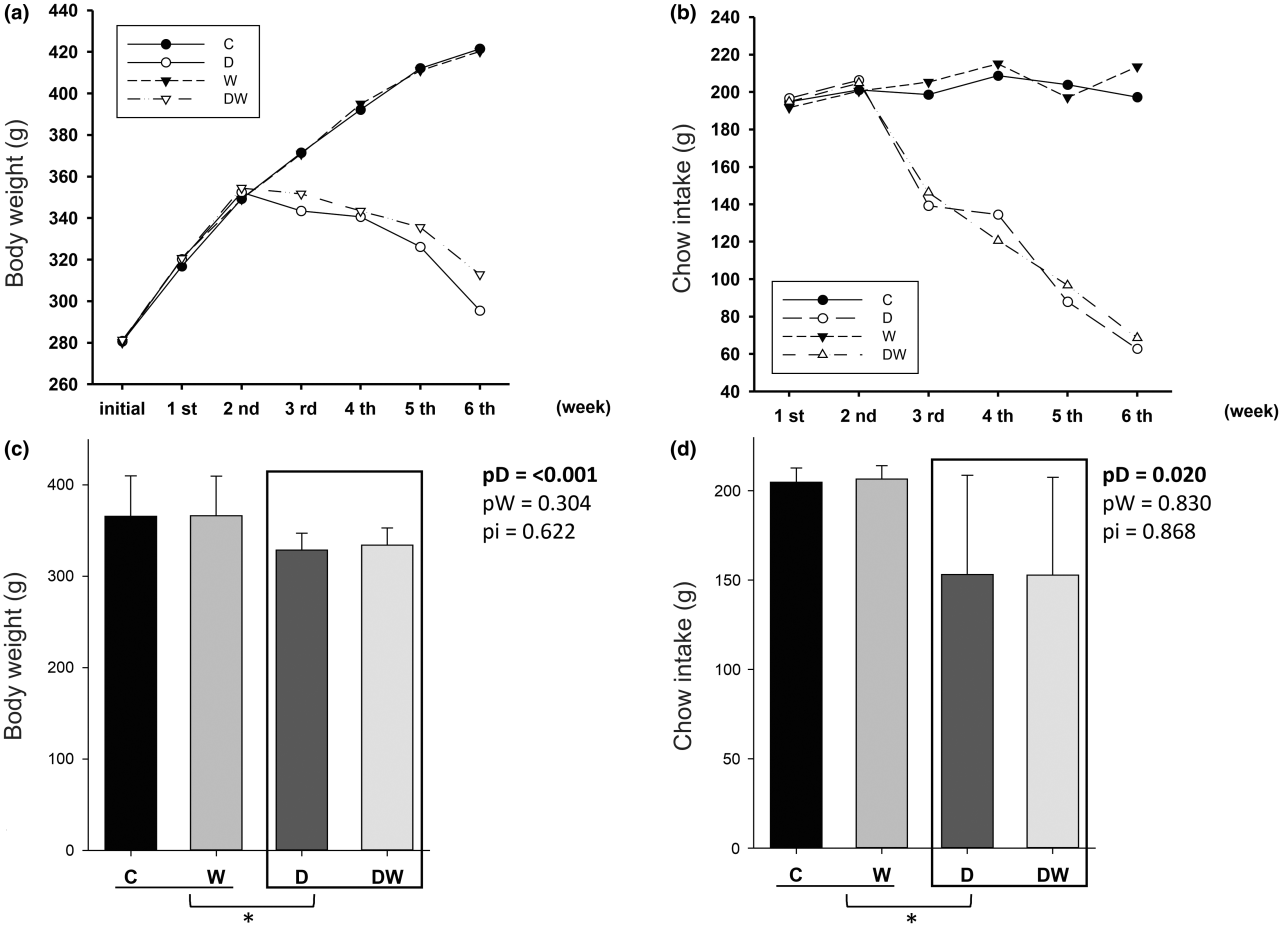
Animal body weight and chow intake evolution (a, b). Area under the curves for body weight and chow intake evolution (c, d). C, control; D, doxorubicin; DW, doxorubicin + Omega‐3 fatty acids. pD, value for doxorubicin effect; pW, value for omega‐3 fatty acid effect; pi, value for doxorubicin + omega‐3 fatty acid interaction; W, omega‐3 fatty acids. Data are expressed as means ± SD. *p*, values are from two‐way analysis of variance. *Animals treated with doxorubicin were statistically different from animals not receiving chemotherapy.

Body weight and chow intake were similar between groups during the initial supplementation phase. Doxorubicin administration was initiated in the third week, and doxorubicin‐treated animals had lower food intake and lost weight compared to animals not receiving chemotherapy (Figure 1a–d).

### 3.2 Cardiac structure and function

Table 1 shows echocardiogram results. Doxorubicin‐treated rats had reduced heart rate compared to non‐treated rats. They also had increased left atrium diameter and LV systolic and diastolic diameter corrected for animal body weight than animals not receiving doxorubicin. Associated doxorubicin + omega‐3 fatty acid treatment improved LV systolic and diastolic diameter corrected for body weight, and atrium diameter corrected for aortic diameter, which suggests that omega‐3 treatment attenuated the doxorubicin‐induced morphological changes. Regarding diastolic function, doxorubicin decreased E wave and prolonged EDT and IVRT, even when IVRT was corrected for heart rate. We observed an improvement in E wave when omega‐3 fatty acids were administered concomitantly with doxorubicin, which together with the reduction in left atrium diameter suggested an improvement in diastolic dysfunction. Doxorubicin worsened S′ wave and LV shortening fraction, and improved LV shortening fraction was seen in DW compared to D, which indicates omega‐3 fatty acids attenuated doxorubicin‐induced systolic dysfunction.

**TABLE 1:** Heart rate, morphological, and cardiac functional variables by echocardiogram.

Variables	C (*n* = 14)	D (*n* = 15)	W (*n* = 14)	DW (*n* = 15)	pD	pW	pi
HR (bpm)	324 ± 57	253 ± 53	292 ± 47	275 ± 77	**0.007**	0.774	0.097
PWT (mm)^a^	1.40 (1.29–1.50)	1.21 (1.13–1.45)	1.44 (1.25–1.53)	1.45 (1.16–1.50)	0.159	0.543	0.559
IVST (mm)^a^	1.34 (1.26–1.50)	1.21 (1.13–1.53)	1.40 (1.26–1.54)	1.50 (1.16–1.53)	0.639	0.620	0.818
LVSD (mm)	3.17 ± 0.90	3.91 ± 0.67	3.34 ± 0.83	3.32 ± 0.72	**0.040**	0.377	0.055
LVSD/BW	7.50 ± 1.82	13.5 ± 2.71	7.94 ± 1.82**	10.6 ± 2.17** ^,^ ***	<0.001	0.043	**0.006**
LVDD (mm)	7.23 ± 0.92	7.26 ± 0.67	7.44 ± 0.67	7.06 ± 0.60	0.368	0.984	0.290
LVDD/BW	17.2 ± 1.85	25.0 ± 3.90*	17.8 ± 1.47	22.7 ± 2.24** ^,^ ***	<0.001	0.354	**0.046**
LAD (mm)	4.96 ± 0.50	5.18 ± 0.30	5.16 ± 0.63	4.75 ± 0.37** ^,^ ***	0.448	0.347	**0.013**
AoD (mm)	3.78 ± 0.15	3.58 ± 0.23	3.77 ± 0.20	3.75 ± 0.20	**0.044**	0.111	0.091
LAD/AoD	1.31 ± 0.11	1.45 ± 0.11*	1.36 ± 0.11	1.27 ± 0.11** ^,^ ***	0.467	0.028	**<0.001**
E (cm/s)	89.7 ± 10.12	66.1 ± 12.73*	86.0 ± 8.89	74.6 ± 13.3** ^,^ ***	<0.001	0.402	**0.036**
A (cm/s)^a^	60.6 ± 14.5	44 (29–72)	55.2 ± 12.1	49.9 ± 13.3	0.336	0.148	0.747
E/A^a^	1.52 (1.30–1.73)	1.42 (0.78–2.41)	1.61 ± 0.30	1.57 ± 0.35	0.556	0.566	0.357
EDT (ms)^a^	46.5 ± 2.17	50.0 (39.5–60.5)	48.0 ± 4.57	49.8 ± 6.56	0.644	0.139	0.950
IVRT (ms)^a^	23 (22–26)	35.5 ± 8.75	24 (22–26)	29.1 ± 5.71	**<0.001**	0.481	0.012
IVRT/RR^a^	55.3 ± 4.99	71.4 ± 14.8	54.8 ± 6.90	61.2 ± 12.7	**<0.001**	0.398	0.026
E′ (cm/s)^a^	4.87 (3.74–6.54)	3.87 ± 0.80	4.96 ± 1.31	4.90 ± 1.29	0.027	0.836	0.012
A′ (cm/s)^a^	3.80 ± 0.10	3.90 (3.55–5.3)	3.94 ± 0.58	3.73 ± 0.59	0.356	0.215	0.220
E/E′^a^	18.6 ± 3.76	17.6 ± 4.43	18.0 (14.7–21.8)	14.5 (13.2–20.2)	0.264	0.765	0.369
LVFS	0.57 ± 0.07	0.46 ± 0.07*	0.56 ± 0.08	0.53 ± 0.08**	0.002	0.164	**0.046**
S′ (cm/s)^a^	4.95 (3.54–5.95)	3.05 (2.75–4.65)	4.57 (3.47–5.85)	3.20 (2.95–5.30)	0.014	0.679	0.337

*Note*: Data are expressed as means ± SD or medians (1st‐3rd quartile). *p* values are from two‐way analysis of variance. In this analysis, if pi < 0.05, we perform the comparison between groups. If pi > 0.05, we analyze separately the effect of each factor (pD or pW). The bold *p* values were considered for the discussion.

Abbreviations: A, peak transmitral flow velocity during atrial contraction; A'm, mean mitral ring displacement in the lateral and septal walls during initial diastole in tissue Doppler; AoD, aortic diameter; BW, body weight (sixth week); C, Control group; D, Doxorubicin group; DW, Doxorubicin + Omega‐3 fatty acids group; E, peak early ventricular filling velocity; EDT, E‐wave deceleration time; E'm, mean mitral ring displacement in the lateral and septal walls during initial diastole in tissue Doppler; HR, heart rate; IVRT/RR, IVRT corrected by heart rate; IVRT, isovolumetric relaxation time; IVST, interventricular septum thickness; LAD, left atrium diameter; LVDD, LV diastolic diameter; LVFS, LV fraction shortening; LVSD, left ventricle (LV) systolic diameters; pD, value for doxorubicin effect; pi, value for doxorubicin + omega‐3 fatty acid interaction; pW, value for omega‐3 fatty acid effect; PWT, posterior wall thickness; S'm, mean mitral ring displacement the in the lateral and septal walls during initial systole in tissue Doppler; W, Omega‐3 fatty acids group.

^a^ Variables with non‐normal distribution were evaluated by the *t* or Mann–Whitney test, and *p* < .008 was considered statistically significant, according to Bonferroni correction.

* Compared to C group.

** *p* < .05 compared to D group.

*** *p* < .05 compared to W group.

### 3.3 Serum lipid profile and serum creatinine

**FIGURE 2:**
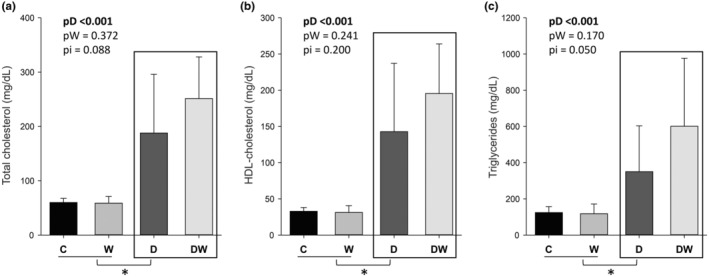
Serum concentration of total cholesterol (a), HDL‐cholesterol (b), and triglycerides (c). C, control; D, doxorubicin; DW, doxorubicin + omega‐3 fatty acids. pD, value for doxorubicin effect; pW, value for omega‐3 fatty acids effect; pi, value for doxorubicin + omega‐3 fatty acids interaction; W, omega‐3 fatty acids. Data are expressed as means ± SD. *p*, values are from two‐way analysis of variance. *Animals treated with doxorubicin were statistically different from animals not treated with chemotherapy.

Doxorubicin‐treated animals showed increased serum total cholesterol, HDL‐cholesterol, and triglycerides; omega‐3 fatty acid supplementation had no effect on these parameters (Figure 2a–c). We did not observe differences in serum creatinine concentration between groups [C: 0.35 (0.32–0.06); D: 0.27 (0.06–0.33); W: 0.38 (0.07–0.02); DW: 0.00 (0.00–0.08) mg/dL; pD = 0.015; pW = 0.615; pi = 0.019 (for this analysis, we considered a statistical significance of *p* < .008)].

### 3.4 Neutral sphingomyelinase activity

**FIGURE 3:**
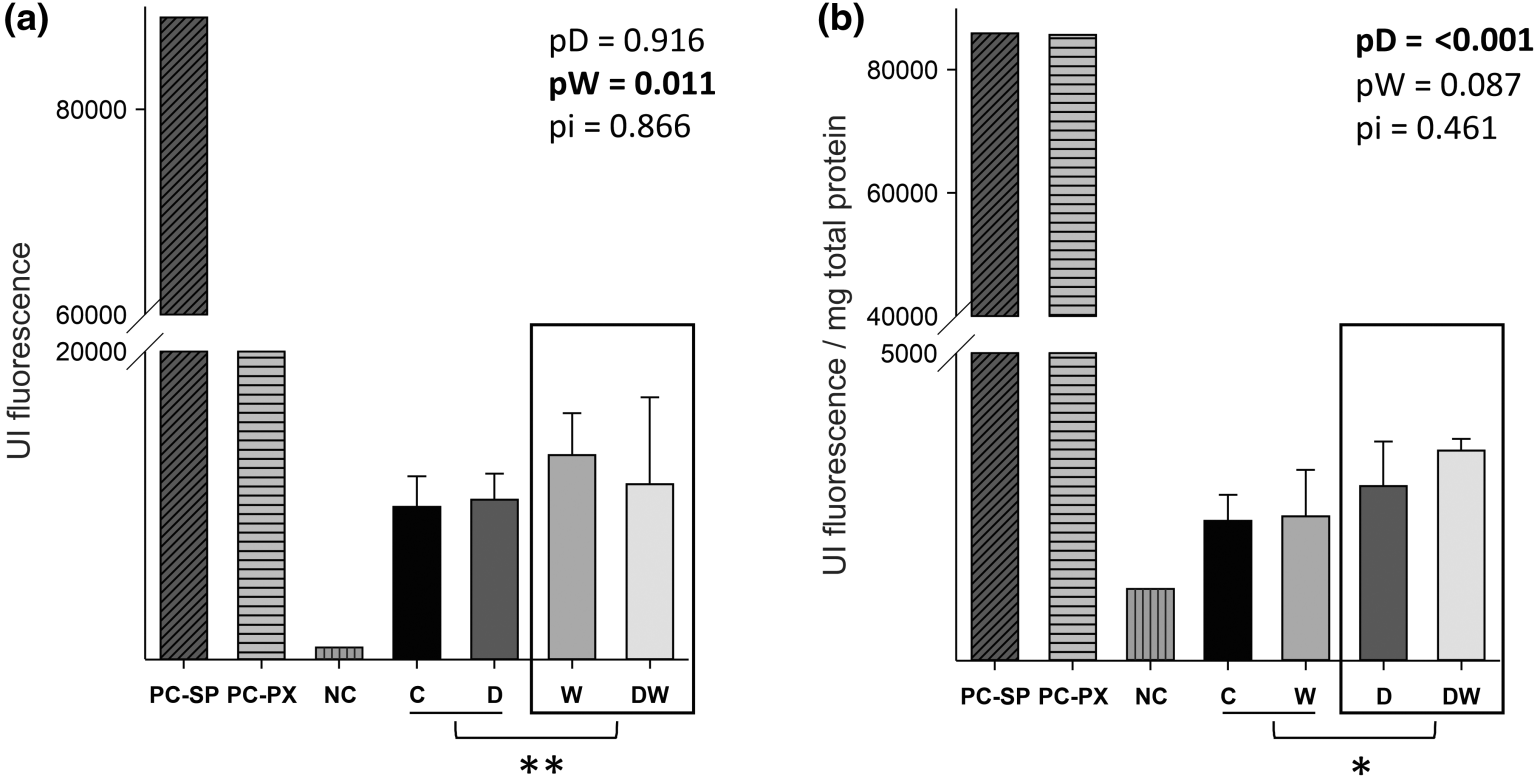
Neutral sphingomyelinase activity in animal serum (a) and neutral sphingomyelinase activity in left ventricle (b). C, control; D, doxorubicin; DW, doxorubicin + omega‐3 fatty acids; NC, negative control; PC‐SP, neutral sphingomyelinase positive control; PC‐PX, peroxidase positive control; pD, value for doxorubicin effect; pW, value for omega‐3 fatty acids effect; pi, value for doxorubicin + omega‐3 fatty acids interaction; W, omega‐3 fatty acids. Data are expressed as means ± SD. *p*, values are from two‐way analysis of variance. *Animals treated with doxorubicin were statistically different from animals not treated with chemotherapy. **Animals treated with omega‐3 fatty acids were statistically different from animals not treated with supplementation.

Animals that received omega‐3 fatty acid supplementation had increased enzymatic activity compared to those that did not (Figure 3a). On the other hand, doxorubicin was responsible for increasing neutral sphingomyelinase enzymatic activity in cardiac tissue compared to animals that did not receive chemotherapy (Figure 3b).

### 3.5 Oxidative stress

**FIGURE 4:**
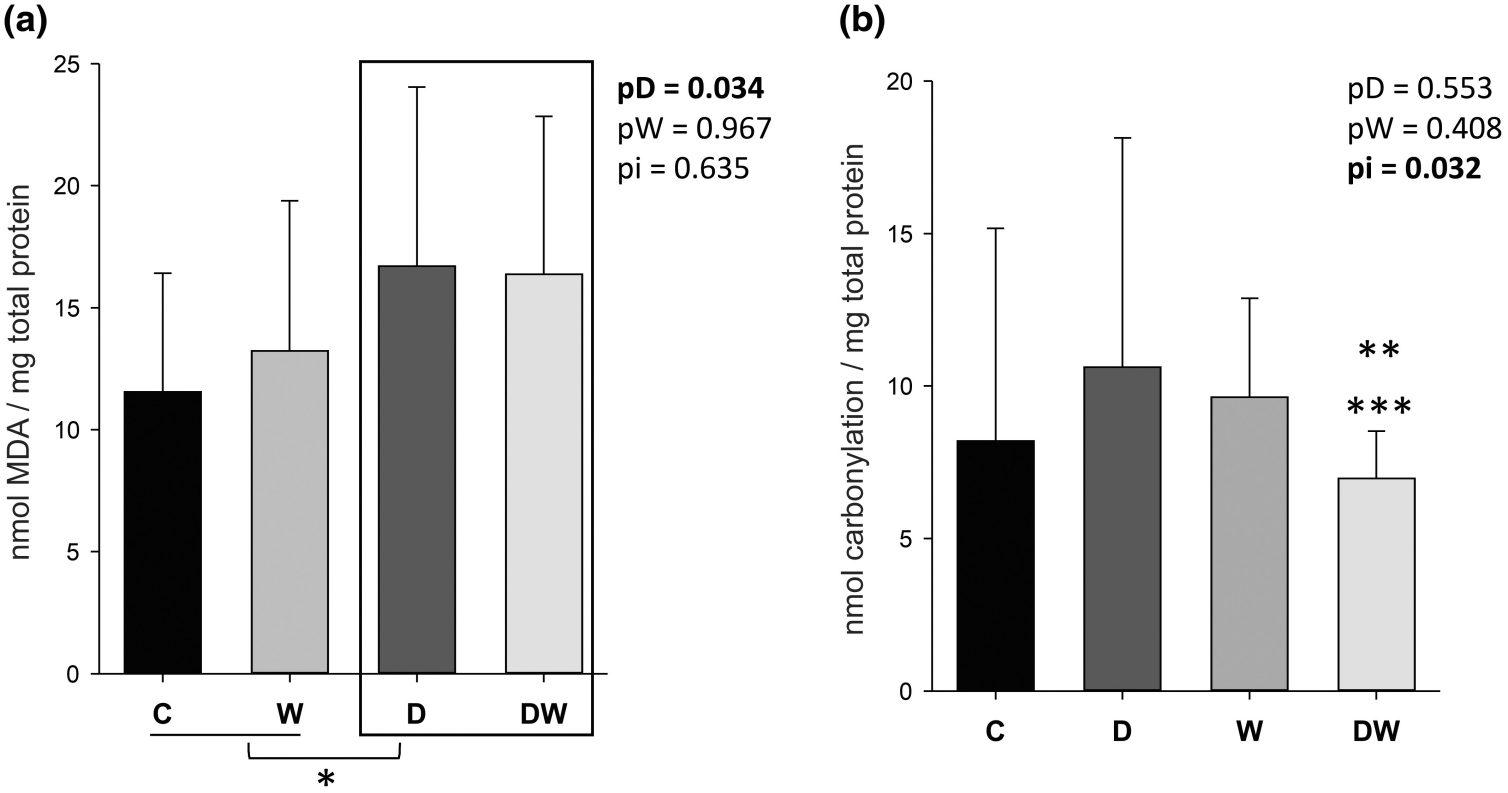
Malondialdehyde concentration in cardiac tissue (a). Carbonylation protein in cardiac tissue (b). C, control; D, doxorubicin; DW, doxorubicin + omega‐3 fatty acids. pD, value for doxorubicin effect; pW, value for omega‐3 fatty acids effect; pi, value for doxorubicin + omega‐3 fatty acids interaction; W, omega‐3 fatty acids. Data are expressed as means ± SD. *p*, values are from two‐way analysis of variance. *Animals treated with doxorubicin were statistically different from animals not treated with chemotherapy. **p < .05 compared to D. ***p < .05 compared to W.

MDA and total protein carbonylation were quantified to assess oxidative stress. We observed that treatment with doxorubicin increased MDA concentration in cardiac tissue (Figure 4a). DW showed lower protein carbonylation concentration than D as shown in Figure 4b.

### 3.6 Quantification of protein in myocardium

**FIGURE 5:**
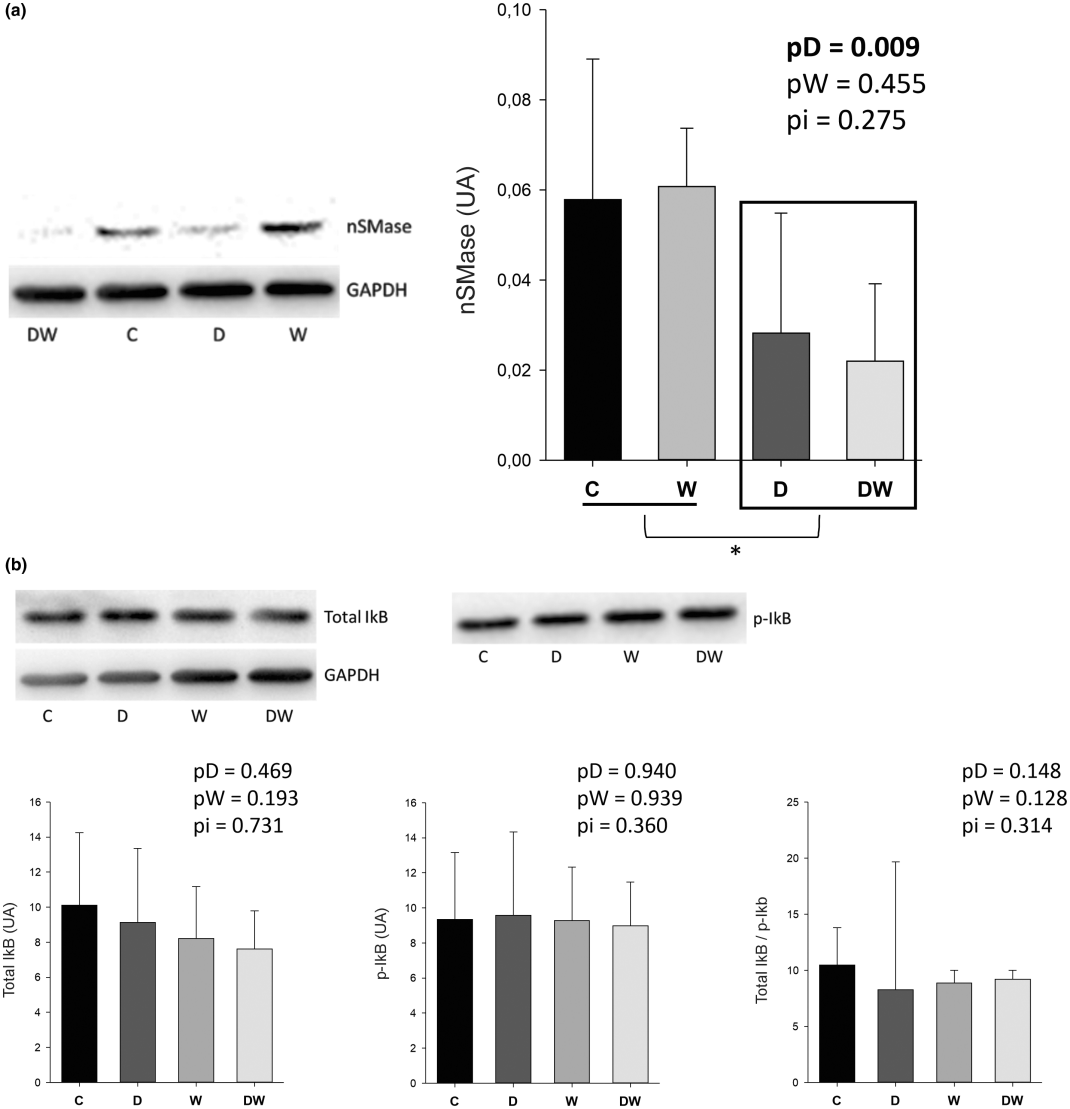
Western blot analysis of nSMase1 expression (a) and total IkB, phosphorylated IkB, and total/phosphorylated ratio (b). C, control; D, doxorubicin; DW, doxorubicin + omega‐3 fatty acids. pD, value for doxorubicin effect; pW, value for omega‐3 fatty acids effect; pi, value doxorubicin + omega‐3 fatty acids interaction; W, omega‐3 fatty acids. Data are expressed as means ± SD. *p*, values are from two‐way analysis of variance. *Animals treated with doxorubicin were statistically different from animals not treated with chemotherapy. IkB and p‐IkB were incubated on the same membrane using stripping technique, and the same GADPH was used to normalize both.

We quantified proteins nSMase1 and IkB in animal cardiac tissue. Doxorubicin‐treated rats showed reduced nSMase1 expression compared to non‐treated animals (Figure 5a). There was no statistical difference between groups for the expression of total IkB, phosphorylated IkB, and total/phosphorylated IkB ratio (Figure 5b).

### 3.7 nSMase1 marker in cardiac tissue sections

**FIGURE 6:**
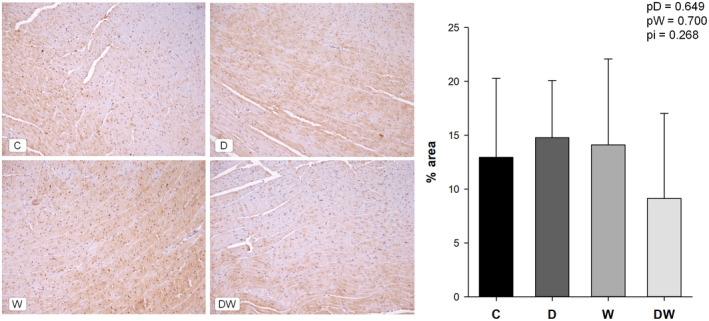
nSMase marked by immunohistochemistry. Images captured under a microscope at 20×. C, control; D, doxorubicin; DW, doxorubicin + omega‐3 fatty acids. pD, value for doxorubicin effect; pW, value for omega‐3 fatty acids effect; pi, value for doxorubicin + omega‐3 fatty acid interaction; W, omega‐3 fatty acids. Data are expressed as means ± SD. *p*, values are from two‐way analysis of variance.

There was no difference between groups for nSMase1 marked area percentage or for its distribution in cardiac tissue (Figure 6).

## 4 DISCUSSION

Doxorubicin is a widely prescribed chemotherapy drug for use in clinical practice because of its high effectiveness in cancer treatment (Yagmurca et al., 2003). However, it can cause toxicity, which is dose‐dependent, in several organs, especially the heart (Vejpongsa & Yeh, 2014). Despite many studies having tried to identify a therapeutic strategy that could attenuate or prevent doxorubicin‐induced cardiotoxicity, there are still no recognized treatments for this condition. Additionally, the aspects of cardiotoxicity pathophysiology are not completely understood (Christidi & Brunham, 2021; Rawat et al., 2021). In this study, doxorubicin caused cardiac structural changes, left ventricular diastolic and systolic dysfunction, and increased oxidative stress. Our results also suggest that the ceramide pathway is involved in doxorubicin‐induced cardiotoxicity. However, the positive effect of omega‐3 fatty acid supplementation was not related to ceramide dynamics, as we had previously hypothesized.

Sphingolipids are bioactive lipids found in cellular plasmatic membranes and are involved in several functions in physiological and pathological processes, including cell survival and oxidative stress regulation. Sphingomyelins are the most abundant sphingolipids present in cells and are composed of ceramides, fatty acid chains, and phosphocholine radicals (Albeituni & Stiban, 2019; Sahu et al., 2019). Some stimuli, such as chemotherapy, can hydrolyze the sphingomyelins by enzymatic reactions and result in ceramide production. The sphingomyelin/ceramide cycle is widely studied for its role in cell differentiation, inflammation, oxidative stress, and apoptosis (Bienias et al., 2016).

Ceramides are synthesized by three pathways: the De Novo pathway, the sphingomyelinase pathway, and the salvage pathway. In the sphingomyelinase pathway, ceramide is formed by hydrolysis of phosphodiester links, which can be mediated by acid sphingomyelinase (aSMase) and neutral sphingomyelinases (nSMase). The latter has a higher association with pathological conditions (de Wit et al., 2020). Four types of nSMases have been identified: nSMase1, nSMase2, nSMase3, and mitochondrial nSMase (Ma‐nSMase) (El Amrousy et al., 2022). The first, nSMase1, is expressed in the heart, although its role in sphingomyelin hydrolysis is unknown. The third, nSMase3, seems to be highly expressed in cardiac tissue and may be important in cardiac function and pathology (Pavoine & Pecker, 2009).

Doxorubicin has previously been shown to promote the activation of sphingomyelinases in cardiac myocytes, increase expression of enzymes such as aSMase in rat heart tissue, and increase ceramide production in both situations (Andrieu‐Abadie et al., 1999; Zhang et al., 2021). Also, some studies have shown that omega‐3 fatty acid supplementation attenuated oxidative stress and apoptosis by reducing ceramide production (Jin et al., 2018; Kasbi‐Chadli et al., 2016). However, in our study, we observed that omega‐3 fatty acid supplementation increased serum nSMase activity and doxorubicin treatment increased nSMase activity and decreased nSMase1 protein content in heart tissue. This divergence between enzyme activity and enzyme expression in animal heart tissue can be explained by the fact that the analysis of enzymatic activity encompasses the activity of all nSMases, and the antibody used to evaluate protein expression was specific for nSMase1. We can therefore suggest that doxorubicin can inhibit nSMase1 expression and at the same time increase the enzymatic activity of other nSMases, such as types 2 and 3. Additionally, the increase in enzymatic activity in rat serum caused by omega‐3 fatty acid treatment suggests that it served as a substrate for the enzymes.

Studies with DHA supplementation and sphingomyelinase expression have described lower aSMase and nSMase expression and activity in human retinal endothelial cells (HREC) treated with IL‐1β and TNF‐α (Opreanu et al., 2010, 2011). However, in our study, omega‐3 fatty acid supplementation did not reduce nSMase activity, which was elevated by doxorubicin in cardiac tissue and contributed to increased enzyme activity in the serum of the animals. This discrepancy may be due to differences in study models, target tissues, supplementation doses, and types of omega‐3 used. It appears that doxorubicin did not affect this inflammatory pathway in our study context, which may explain the observed ineffectiveness of omega‐3 fatty acid supplementation.

TNF‐α is essential in nSMase activation and is responsible for degrading protein IkB. When IkB binds to transcription factor NF‐kB, the function of NF‐kB is inhibited. When IkB is degraded, NF‐kB activation occurs triggering inflammation and apoptosis (Karin, 1999). Wang et al. (2016) observed decreased total/phosphorylated IkB ratio and total/phosphorylated NF‐kB ratio in cells which received acute treatment with doxorubicin and DHA compared to cells that received doxorubicin alone. In our study, there was no statistical difference in IkB expression between groups, not even in doxorubicin‐treated animals that showed an increase in nSMase activity. This divergence between results could be explained by DOX administration and the use of isolated DHA.

Omega‐3 fatty acid supplementation seems to reduce serum triglycerides in people with severe hypertriglyceridemia ≥500 mg/dL (Li et al., 2019), and administration of highly purified EPA associated with statin has been shown to reduce mortality and cardiovascular events in people with mild‐to‐moderate hypertriglyceridemia (between 135 and 499 mg/dL) (Bhatt et al., 2019). Epidemiological studies have also shown a relationship between a reduction in cardiovascular events and omega‐3 fatty acid consumption (Del Gobbo et al., 2016; García‐Alonso et al., 2012). On the other hand, clinical trials with a cardiovascular disease (heart failure, acute myocardial infarction, or vascular atherosclerosis disease) or diabetes mellitus population did not show beneficial effects from omega‐3 fatty acid supplementation (Bowman et al., 2018; Kromhout et al., 2010; Tavazzi et al., 2008).

Studies with doxorubicin‐induced cardiotoxicity and omega‐3 fatty acid supplementation have given controversial results (Serini et al., 2017). In vivo and in vitro experimental studies describe hyperlipidemia as a side effect of doxorubicin administration (Bizzi et al., 1983; Klinnikova et al., 2016; Korga et al., 2013; Zhang & Li, 2019). Increases in serum HDL, LDL, total cholesterol, and triglycerides were reported 14 days after chemotherapy administration. This change was associated with the development of nephrotic syndrome – kidney damage characterized by proteinuria, hypoalbuminemia, edema, and hyperlipidemia – which can be caused by doxorubicin (Agrawal et al., 2018). Animals in our study that received doxorubicin showed increased serum HDL, total cholesterol, and triglyceride concentrations. However, no differences were observed in serum creatinine concentrations between groups. These results suggest that dyslipidemia associated with doxorubicin administration may have different etiologies and not just result from chemotherapy drug‐induced kidney injury. The harmful consequences of these changes in blood lipids, such as cardiac lipotoxicity, still require investigation in this model. In this context, a study that evaluated omega‐3 fatty acid supplementation in doxorubicin‐induced nephrotoxicity showed improved lipid profile and serum creatinine in rats that received associated treatment (Saleh et al., 2020). However, the doses used in the study were 25, 50, and 100 mg/kg/day, which are quite different from our study.

Oxidative stress has been the focus of most studies into doxorubicin‐induced toxicity. The heart is considered a target organ due to the abundance of mitochondria and cardiolipin and low antioxidant enzyme concentrations compared with other organs (Songbo et al., 2019). Increases in oxidative stress can damage cellular compounds, including lipids in cell membranes, proteins, and DNA. Lipid peroxidation increases myocardial MDA concentration, as shown in rats from our study that received doxorubicin. Previous studies have evaluated the effect of omega‐3 fatty acid supplementation in doxorubicin‐induced cardiotoxicity; they observed decreases in MDA concentration in the presence of omega‐3 fatty acid supplementation (Saleh et al., 2020; Uygur et al., 2014; Yu et al., 2013). However, only one was performed in a chronic cardiotoxicity model (Yu et al., 2013), and omega‐3 fatty acid supplementation varied between studies. In agreement with our findings, another study with chronic cardiotoxicity and omega‐3 fatty acid supplementation (370 mg IP on alternate days) did not find changes in MDA concentration (Xue et al., 2016). However, El Amrousy et al. (2022) observed a decrease in serum MDA concentration in children with leukemia who were treated with doxorubicin plus high‐dose omega‐3 fatty acid supplementation (dose of 1000 mg/day).

Carbonylation is widely used to evaluate oxidative damage to proteins. The introduction of carbonyl groups in specific amino acids of a protein structure marks the protein degradation through oxidative reaction (Nyström, 2005). Additionally, protein carbonylation could be induced by the link between proteins and reactive aldehydes originating from lipid peroxidation or by reducing sugars. These proteins can affect important cellular signaling pathways such as apoptosis and oxidative stress. Although the chemistry involved in this process is well established, the modifications generated by protein carbonylation are still not fully understood (Akagawa, 2021). We did not identify any other studies that evaluated protein carbonylation in doxorubicin‐induced cardiotoxicity associated with omega‐3 fatty acid treatment.

Our DW group showed a decrease in total protein carbonylation compared to our D group, suggesting an antioxidant effect from omega‐3 fatty acids. Considering that reactive aldehydes MDA, 4‐hydroxy‐2‐nonenal (HNE), 4‐oxo‐2‐nonenal, and acrolein resulting from lipid peroxidation are responsible for carbonyl proteins, we can suggest that the increase in protein carbonylation in DW may be associated with less aldehyde formation which was not evaluated in this study. Also, we can specifically suggest that there was an increase in HNE aldehyde by lipid peroxidation; this is considered the most cytotoxic aldehyde due to its ease of movement into and out of cells allowing the alteration of long proteins at the site of the originating event (Akagawa, 2021). This resulting HNE could reside in the carbonylated proteins outside the heart in the associated treatment condition (DW group).

Regarding cardiac function, studies on acute doxorubicin treatment and omega‐3 fatty acid supplementation in rats have seen improvements in cardiac function (Saleh et al., 2020; Schjøtt et al., 1996). Studies with chronic doxorubicin treatment and omega‐3 fatty acid supplementation have had divergent results (Carbone et al., 2012; El Amrousy et al., 2022; Xue et al., 2016; Yu et al., 2013). Our study showed improvement in left ventricular systolic function and a slight improvement in left ventricular diastolic function.

An important determinant of cardiac function is volemic status which is related to preload and afterload (Santos et al., 2014). In our study, we observed decreases in body weight and food intake which have already been seen in other studies (Carbone et al., 2012; Dantas et al., 2022), and which could be associated to dehydration (Pugazhendhi et al., 2018). However, in a dehydration state we expected decreases in cardiac chambers (de Carvalho et al., 2016), the opposite of our findings. For this reason, we believe that the cardiac dysfunction observed in our study is directly related to cardiotoxicity.

Finally, considering the positive effect of omega‐3 fatty acid supplementation in cardiac function and oxidative stress markers in this chronic model of doxorubicin‐induced cardiotoxicity, and its intake safety, omega‐3 fatty acid supplementation is a good therapeutic option for testing in future clinical trials.

## 5 CONCLUSION

Omega‐3 fatty acid supplementation was able to attenuate cardiac dysfunction and oxidative protein damage induced by doxorubicin. Although the ceramide pathway is possibly involved in the pathophysiology of cardiotoxicity, the omega‐3 fatty acid was unable to decrease the nSMase activity increased by doxorubicin in cardiac tissue and it is not responsible to alter the nSMase1 expression too. Therefore, sphingomyelin/ceramide pathway is not the mechanism by which omega‐3 fatty acids attenuated doxorubicin‐induced cardiotoxicity.

## AUTHOR CONTRIBUTIONS

**Marina Gaiato Monte:** Conceptualization (supporting); formal analysis (equal); investigation (lead); methodology (supporting); project administration (lead); visualization (equal); writing – original draft (equal); writing – review and editing (equal). **Carolina Rodrigues Tonon:** Visualization (equal); writing – review and editing (supporting). **Anderson Seiji Fujimori:** Investigation (supporting). **Ana Paula Dantas Ribeiro:** Investigation (supporting). **Silmeia Garcia Zanati:** Investigation (supporting). **Katashi Okoshi:** Investigation (supporting). **Camila Renata Correa Camacho:** Investigation (supporting). **Maria Regina Moretto:** Investigation (supporting). **Sergio Alberto Rupp de Paiva:** Formal analysis (supporting); writing – review and editing (supporting). **Leonardo Antonio Mamede Zornoff:** Writing – review and editing (supporting). **Paula Schmidt Azevedo:** Writing – review and editing (supporting). **Marcos Ferreira Minicucci:** Writing – review and editing (supporting). **Bertha Furlan Polegato:** Conceptualization (lead); formal analysis (equal); funding acquisition (lead); investigation (supporting); methodology (lead); project administration (supporting); supervision (lead); writing – original draft (equal); writing – review and editing (equal).

## FUNDING INFORMATION

Research funded by CNPq (Conselho Nacional para o Desenvolvimento Científico e Tecnológico, Process no. 407201/2021‐1) and FAPESP (Fundação de Amparo à Pesquisa do Estado de São Paulo, Process no. 2022/15954‐9). Scholarship funded by FAPESP (Process no. 2018/25677‐7).

## CONFLICT OF INTEREST STATEMENT

The authors declare that they do not have any conflict of interest.

## Data Availability

The data that support the findings of this study are available from the corresponding author upon reasonable request.
